# Information Theory and Language

**DOI:** 10.3390/e22040435

**Published:** 2020-04-11

**Authors:** Łukasz Dębowski, Christian Bentz

**Affiliations:** 1 Institute of Computer Science, Polish Academy of Sciences, ul. Jana Kazimierza 5, 01-248 Warszawa, Poland; 2 URPP Langauge and Space, University of Zürich, Freierstrasse 16, CH-8032 Zürich, Switzerland; chris@christianbentz.de; 3 DFG Center for Advanced Studies, University of Tübingen, Rümelinstraße, D-72070 Tübingen, Germany

**Keywords:** entropy, mutual information, natural language, statistical language models, statistical language laws, semantics, syntax, complexity, criticality, language resources

Human language is a system of communication. Communication, in turn, consists primarily of information transmission. Writing about the interactions between information and natural language, we cannot fail to mention that information theory has originated with statistical investigations of English text in the turn of the 1940s and 1950s [1,2]. While initially, there were some common interests between information theory and linguistics, for instance, understanding distributional properties of elements in natural language, e.g., [3,4], the following decades brought a growing divide between the fields. They went down separate research paths until the end of the 20th century. Whereas information theory embraced probabilities, also in disguise of algorithms [5], the influential Chomskyan formal theory of syntax deemed the question of probabilities in language as scientifically largely irrelevant [6]. It was only in the 1990s that the gap between information theory and formal language studies started to be bridged by the rapid progress of computational linguistics [7,8]. For a detailed account of this development see also [9]. Presently, this progress has resulted in large-scale neural statistical language models such as the much publicized GPT-2 [10], which is capable of generating surreal but understandable short stories.

To use an information theoretic metaphor, the communication channel between the divergent research traditions is reopening. Looking back at independent discoveries of probabilistic and non-probabilistic accounts of natural language, we deem that the divide might have been necessary to focus attention on particular areas of scientific investigation. However, the time is ripe to integrate the established disjoint scholarships, and to cross-fertilize research. We believe that the frameworks of information theory and linguistics are fully compatible in spite of some historical reservations and different academic curricula.

This Special Issue consists of twelve contributions that cover various recent research areas at the interface of information theory and linguistics. They concern in particular:

- applications of information theoretic concepts to the research of natural languages;
- mathematical work in information theory inspired by natural language phenomena;
- empirical and theoretical investigation of quantitative laws of natural language;
- empirical and theoretical investigation of statistical and neural language models.

We believe that the selection of authors and topics in this Special issue reflects the state of the art of interdisciplinary research. In fact, the formal disciplines of the contributing authors range from linguistics and cognitive science to computer science, mathematics, and physics. Since the various research perspectives cannot be easily arranged in an obvious linear order, we have decided to present the papers in the order of their publication.

## The Contributions

- Koplenig, A., Wolfer, S., and Müller-Spitzer, C., *Studying Lexical Dynamics and Language Change via Generalized Entropies: The Problem of Sample Size* [11].

Dependence on sample size is a recurrent problem in quantitative linguistics. This also holds for accounts harnessing, for instance, the entropy of word frequency distributions. Koplenig, Wolfer, and Müller-Spitzer systematically investigate this issue based on a corpus compiled from a weekly news magazine in German, which spans seven decades, and contains more than 200 million word tokens. In particular, they employ the generalized Tsallis entropies, which allow for weighting parts of the frequency spectrum more or less heavily in entropy calculations. It turns out that correlations between the estimated entropies and respective sample sizes are only broken if a heavy bias towards highly frequent words is introduced. In particular, the standard Shannon entropies display a strong dependence on sample size. In an application investigating lexical change over several decades, the authors further propose and illustrate a “litmus test”. This entails calculating entropy divergences between parts of the corpus over historical time, and comparing these with entropy divergences calculated for texts in random order. Their results suggest that it is the growing sample size over time which leads to systematic patterns in entropy divergences, potentially independent of genuine lexical change.

- Hahn, M. and Futrell, R., *Estimating Predictive Rate–Distortion Curves via Neural Variational Inference* [12].

The predictive rate-distortion curve quantifies the trade-off between compressing information about the past of a stochastic process and predicting its future accurately. Hence it is a more detailed characteristic of the process complexity than its excess entropy or statistical complexity. Hahn and Futrell study estimation of predictive rate-distortion curves for complex stochastic processes, aimed to be applied for natural language. The authors’ method of estimation consists in upper bounding the correct curve by means of a neural network approximation of the investigated process. The method is validated on examples of processes for which the predictive rate-distortion curve is known analytically. Moreover, the authors provide an estimate of the predictive rate-distortion curve for text corpora in five natural languages (English, Russian, Arabic, Japanese, and Chinese). The experiments universally indicate that the excess entropy and statistical complexity for natural language are infinite.

- Hernández-Fernández, A., Torre, I.G., Garrido, J.M., and Lacasa, L., *Linguistic Laws in Speech: The Case of Catalan and Spanish* [13].

There is a hypothesis in quantitative linguistics, called the physical hypothesis, that statistical linguistic laws in written texts are a byproduct of more exact laws present in the acoustic signals of oral communication. In contrast to earlier works, Hernández-Fernández et al. investigate and verify the physical hypothesis using a large oral text corpus, the Glissando Corpus of spoken Catalan and Spanish. The studied quantitative linguistic laws include Zipf’s law, Herdan’s law, the brevity law, Menzerath–Altmann’s law, the log-normality law, and the size-rank law. By aligning the acoustic signal with the speech transcripts, they measure and compare the agreement of each of these laws when measured in both physical and symbolic units. The conclusion of this experiment is that quantitative linguistic laws are satisfied indeed more accurately for the acoustic signal than for the speech transcript.

- Venhuizen, N.J., Crocker, M.W., and Brouwer, H., *Semantic Entropy in Language Comprehension* [14].

The link between information and meaning has been a controversial topic ever since Shannon’s work. The alleged disconnection between the two was posed as a main argument against analyzing natural language in the light of information theory. Venhuizen, Crocker and Brouwer illustrate that information theoretic concepts might be fruitfully applied to both linguistic signals, and the points they denote in meaning space. In their experiments, they combine formal semantic tools with neural network technology. Based on a set of training sentences, their neural network learns to map linguistic signals onto meaning vectors representing propositional truth values. This setup allows the authors to trace the semantic expectations of the network in word-by-word online processing. In this context, they tease apart surprisal and entropy reduction, two concepts which were previously often seen as strongly intertwined. Surprisal is calculated based on word-by-word transitions in meaning space, whereas entropy is calculated over meaning vectors which identify a unique semantic model of the world. Given these definitions, suprisal and entropy reduction are not strongly correlated. The authors explain this by pointing out that surprisal is inherently more sensitive to frequency effects in the linguistic signal, while entropy reduction is more strongly influenced by knowledge of the model theoretic world.

- Ren, G., Takahashi, S., and Tanaka-Ishii, K., *Entropy Rate Estimation for English via a Large Cognitive Experiment Using Mechanical Turk* [15].

The entropy rate of a sequence reflects the amount of information conveyed per unit, e.g., characters or words in natural language. It has been proposed also as a measure of the complexity of a sequence. However, estimating the entropy rate of natural languages has proven a challenging endeavor due to the problem of finite sample sizes and long-range dependence. Ren, Takahashi, and Tanaka-Ishii revive an idea going back to Shannon’s experiments [2], namely, estimating the entropy rate by using human subjects to predict the next character in a linguistic sequence. They collect more than 100,000 character predictions for English texts by 683 different subjects. Across all subjects and trials, they estimate the entropy rate to around 1.4 bits per character. Using trials selected for high performance (i.e., correctly guessing characters) reduces the estimate to around 1.22 bits per character. In their discussion, the authors point out that this is lower than Shannon’s original value of 1.3 bits per character. On the other hand, it is higher than entropy rates estimated with current state-of-the-art neural language models, which are just above 1 bit per character. This suggests that neural language models outperform human subjects in character guessing games.

- Gutierrez-Vasques, X. and Mijangos, V., *Productivity and Predictability for Measuring Morphological Complexity* [16].

There is a recent rise of interest in measuring the morphological complexity of typologically diverse languages. The findings of this research have implications for both theoretical and applied linguistics, especially in the domain of natural language processing. Gutierrez-Vasques and Mijangos propose to apply the information-theoretic concept of entropy rate to word internal structure. Their data sets contain parallel texts for 47 and 133 typologically diverse languages respectively. Using a neural language model they estimate the difficulty of predicting character unigrams and trigrams within words for different languages and writing systems. These estimates are then contrasted with more traditional measures of morphological complexity, such as the type-token ratio for words. It turns out that word internal predictability is only weakly correlated with the type-token ratio, and hence measures a new and independent dimension of morphological complexity.

- Dębowski, Ł., *Approximating Information Measures for Fields* [17].

Motivated by some theoretical problems of statistical modeling of natural language, Dębowski reconsiders the classical problem of generalizing entropy and mutual information from discrete random variables (finite partitions, in more abstract formulation) to arbitrary random variables (fields and *σ*-fields, respectively). Having noticed a mistake in his paper from 2009, he supplies corrected proofs of the invariance of completion and the chain rule for conditional entropy and mutual information. In the final section, he also discusses how the generalized calculus of conditional entropy and mutual information is useful in particular for studying the ergodic decomposition of strongly non-ergodic stationary processes and its links with statistical modeling of natural language, which possibly should be modeled by a strongly non-ergodic process.

- Linke, M. and Ramscar, M., *How the Probabilistic Structure of Grammatical Context Shapes Speech* [18].

Frequencies of occurrence are a central concept in quantitative linguistics. They are often used to measure the informativeness of units (i.e., characters, words, etc.) in written language. Linke and Ramscar point out several caveats with this approach. Firstly, written language is not a direct reflection of speech. As a remedy, they use a corpus of conversational English of more than 200,000 word tokens with phonetic labels, and compare their results to studies using written language. Secondly, frequencies of occurrence abstract away from co-occurrence patterns at different levels of language structure, e.g., n-grams for words and parts-of-speech, as well as subword structure. The authors argue that grammatical context often predicts usage patterns in speech better than mere frequencies. Thirdly, distributions of frequencies are mostly analyzed over entire texts, for instance, when power law like patterns such as Zipf’s law are assessed. However, the authors illustrate that there are systematic differences between the distributions of frequencies for words of different parts-of-speech. Namely, while open class items such as nouns and verbs follow power laws, function words rather follow geometric distributions. In fact, the authors further argue that power law like behavior in aggregate distributions might well be the outcome of mixing distributions which are by themselves geometric.

- Gerlach, M. and Font-Clos, F., *A Standardized Project Gutenberg Corpus for Statistical Analysis of Natural Language and Quantitative Linguistics* [19].

Studies on information theoretic properties of natural languages—and analyses in quantitative linguistics more generally—stand and fall with availability of textual data. The universality of linguistic laws, for instance, can only be ascertained given openly available, cross-linguistic, and transparently processed data. To this end, Gerlach and Font-Clos contribute a standardized version of the Project Gutenberg Corpus, which contains more than 50,000 books in over 20 languages. They give a detailed description of the data acquisition, processing, and metadata annotation procedures. Furthermore, they illustrate how this corpus can be used to measure the topical variability between texts associated with different genres via so-called “bookshelf” labels, and how authors are distinguishable by the Jensen-Shannon divergence applied to their works.

- Seoane, L.F. and Solé, R., *Criticality in Pareto Optimal Grammars?* [20].

Seoane and Solé propose a computational methodology to inspect corpora of texts in order to extract salient levels of linguistic description. Their methodology is grounded in the bottleneck method from information theory, Pareto optimality from multi-objective optimization, and concepts from statistical physics such as energy, entropy, phase transitions and criticality. Their working example concerns extracting the Pareto optimal grammars from 49 newspaper articles taken from the Corpus of Contemporary American English preprocessed by the Natural Language Toolkit (NLTK). The numerical results indicate a critical point in the description of human language. As the authors write, the critical point is the worst case in terms of description since there is no relatively small model which can capture the whole phenomenology at any level of linguistic description.

- Ahmadi, L. and Ward, M.D., *Asymptotic Analysis of the kth Subword Complexity* [21].

The subword complexity is a function which counts how many distinct substrings of a given length appear in a given string. It is a simple characteristic of a string that yields an insight whether the string is periodic, random, or something in between—like a text in natural language. In particular, the subword complexity divided by the string length equals to the type-token ratio investigated in quantitative linguistics. Ahmadi and Ward study some properties of subword complexity from a mathematical point of view. Namely, they investigate the asymptotic behavior of the subword complexity for sequences of independent identically distributed random variables. They derive expressions for the expectation (first moment) and the variance (second moment) of subword complexity. Their methodology involves complex analysis, analytical poissonization and depoissonization, the Mellin transform, and saddle point analysis.

- Corral, Á. and Serra, I., *The Brevity Law as a Scaling Law, and a Possible Origin of Zipf’s Law for Word Frequencies* [22].

Corral and Serra study the joint distribution of lengths and frequencies of words, whose marginals are described by the brevity law and Zipf’s law for frequencies of frequencies, called also Lotka’s law. The investigated corpus is the English subcorpus of the Standardized Project Gutenberg Corpus, introduced in contribution [19]. The authors observe that the marginal distribution of word length is better described by the gamma distribution than by the previously proposed log-normal distribution. Moreover, the conditional frequency distributions at a fixed length exhibit a universal power-law decay and a scaling law analogous to those found in the thermodynamics of critical phenomena. In conclusion, the authors present a four-parameter model for the joint distribution of lengths and frequencies of words.

## References

[1] Shannon C. (1948). A mathematical theory of communication. Bell Syst. Tech. J..

[2] Shannon C. (1951). Prediction and entropy of printed English. Bell Syst. Tech. J..

[3] Harris Z. (1968). Mathematical Structures of Language.

[4] Harris Z. (1991). A Theory of Language and Information: A Mathematical Approach.

[5] Kolmogorov A.N. (1965). Three approaches to the quantitative definition of information. Probl. Inf. Transm..

[6] Chomsky N. (1957). Syntactic Structures.

[7] Jelinek F. (1997). Statistical Methods for Speech Recognition.

[8] Manning C.D., Schütze H. (1999). Foundations of Statistical Natural Language Processing.

[9] Pereira F. (2000). Formal grammar and information theory: together again?. Philos. Trans. R. Soc. Lond. Ser. A Math. Phys. Eng. Sci..

[10] Radford A., Wu J., Child R., Luan D., Amodei D., Sutskever I. Language Models are Unsupervised Multitask Learners. https://d4mucfpksywv.cloudfront.net/better-language-models/language-models.pdf.

[11] Koplenig A., Wolfer S., Müller-Spitzer C. (2019). Studying Lexical Dynamics and Language Change via Generalized Entropies: The Problem of Sample Size. Entropy.

[12] Hahn M., Futrell R. (2019). Estimating Predictive Rate–Distortion Curves via Neural Variational Inference. Entropy.

[13] Hernández-Fernández A., Torre I.G., Garrido J.M., Lacasa L. (2019). Linguistic Laws in Speech: The Case of Catalan and Spanish. Entropy.

[14] Venhuizen N.J., Crocker M.W., Brouwer H. (2019). Semantic Entropy in Language Comprehension. Entropy.

[15] Ren G., Takahashi S., Tanaka-Ishii K. (2019). Entropy Rate Estimation for English via a Large Cognitive Experiment Using Mechanical Turk. Entropy.

[16] Gutierrez-Vasques X., Mijangos V. (2019). Productivity and Predictability for Measuring Morphological Complexity. Entropy.

[17] Dębowski Ł. (2020). Approximating Information Measures for Fields. Entropy.

[18] Linke M., Ramscar M. (2020). How the Probabilistic Structure of Grammatical Context Shapes Speech. Entropy.

[19] Gerlach M., Font-Clos F. (2020). A Standardized Project Gutenberg Corpus for Statistical Analysis of Natural Language and Quantitative Linguistics. Entropy.

[20] Seoane L.F., Solé R. (2020). Criticality in Pareto Optimal Grammars?. Entropy.

[21] Ahmadi L., Ward M.D. (2020). Asymptotic Analysis of the kth Subword Complexity. Entropy.

[22] Corral A., Serra I. (2020). The Brevity Law as a Scaling Law, and a Possible Origin of Zipf’s Law for Word Frequencies. Entropy.

